# A rare case of esophageal metastasis from pancreatic ductal adenocarcinoma: a case report and literature review

**DOI:** 10.18632/oncotarget.18458

**Published:** 2017-06-12

**Authors:** Lauren M. Rosati, Megan N. Kummerlowe, Justin Poling, Amy Hacker-Prietz, Amol K. Narang, Eun J. Shin, Dung T. Le, Elliot K. Fishman, Ralph H. Hruban, Stephen C. Yang, Matthew J. Weiss, Joseph M. Herman

**Affiliations:** ^1^ Department of Radiation Oncology & Molecular Radiation Sciences, The Sol Goldman Pancreatic Cancer Research Center, The Johns Hopkins University School of Medicine, Baltimore, MD, USA; ^2^ Department of Pathology, The Sol Goldman Pancreatic Cancer Research Center, The Johns Hopkins University School of Medicine, Baltimore, MD, USA; ^3^ Department of Gastroenterology, The Johns Hopkins University School of Medicine, Baltimore, MD, USA; ^4^ Department of Oncology, The Sol Goldman Pancreatic Cancer Research Center, The Johns Hopkins University School of Medicine, Baltimore, MD, USA; ^5^ Department of Radiology, The Sol Goldman Pancreatic Cancer Research Center, The Johns Hopkins University School of Medicine, Baltimore, MD, USA; ^6^ Department of Surgery, The Sol Goldman Pancreatic Cancer Research Center, The Johns Hopkins University School of Medicine, Baltimore, MD, USA; ^7^ Department of Radiation Oncology, The University of Texas MD Anderson Cancer Center, Houston, TX, USA

**Keywords:** pancreatic cancer, pancreatic ductal adenocarcinoma, metastatic, esophagus, esophageal metastasis

## Abstract

**Purpose:**

We report a very unique case of an esophageal metastasis from a pancreatic ductal adenocarcinoma (PDAC) primary.

**Methods:**

After obtaining consent from the patient, all relevant records of the case were obtained and retrospectively reviewed.

**Results:**

At presentation, the patient was diagnosed with synchronous pancreatic and esophageal cancer. He received six months of neoadjuvant therapy including FOLFIRINOX (5-fluorouracil, leucovorin, irinotecan, and oxaliplatin) and stereotactic body radiation therapy (SBRT) to the pancreatic tumor followed by a combined pancreaticoduodenectomy and Ivor Lewis esophagectomy. Review of the final esophageal specimen revealed normal overlying squamous mucosa with an underlying focus of metastatic PDAC. The patient remains alive with no evidence of disease 17 months from surgery and 25 months from diagnosis.

**Conclusions:**

Differentiating an esophageal metastasis from a PDAC primary versus a synchronous esophageal carcinoma is very difficult despite state-of-the-art diagnostic techniques performed at a high-volume tertiary cancer center. Extensive evaluation and continued follow-up of PDAC patients presenting with a synchronous esophageal lesion in a multidisciplinary setting may help ensure efficient and accurate management. In our case, neoadjuvant FOLFIRINOX and SBRT to the primary PDAC tumor followed by surgery has been an effective approach for this patient.

## INTRODUCTION

Pancreatic ductal adenocarcinoma (PDAC) is the third leading cause of death by a solid malignancy in the United States, with a 5-year overall survival rate of 8%. [1] PDAC is highly aggressive and often diagnosed at an advanced stage due to the inability to detect early symptoms. An autopsy series reported that distant metastasis occurs late during the genetic evolution of PDAC, with an estimated half-decade required for a PDAC to acquire metastatic ability. [2]

PDAC most commonly metastasizes to lymph nodes, the liver, lung, and peritoneal cavity, while rare locations that have been reported include bone, brain, myocardium, and the umbilicus. [3, 4] At this time, there are few known cases of isolated esophageal metastasis from a pancreatic primary. In general, metastases to the esophagus are extremely rare, with rates ranging from 4-11% in patients with primaries of the lung, breast, and stomach. [5, 6]

Not only is a PDAC metastasis to the esophagus extremely rare, but it is also difficult to distinguish an esophageal primary from a metastasis to the esophagus by radiographic imaging or endoscopy. To our knowledge, we report the 2^nd^ case of a metastasis to the esophagus arising from a PDAC primary reported in the modern era (since the 1980s). [7-13]

## RESULTS

### Clinical presentation and treatment recommendations

A 72-year-old non-smoking male presented with a 6-month history of weight loss (9 kg) followed by obstructive jaundice characterized by a 2-month history of acholic stools and dark urine. Past medical history was significant for hypertension and dyslipidemia and an extensive family history of cancer was significant for pancreas, liver, breast, gynecologic, and colon malignancies in 5 siblings and his father. Initial evaluation was conducted by his primary care provider and included laboratory studies and imaging. Computed tomography (CT) scan of the abdomen and pelvis revealed a 2.5 x 1.7 cm mass in the pancreatic head, abutment of the superior mesenteric artery (SMA) and vein (SMV), and marked biliary and pancreatic ductal dilatation consistent with PDAC. Liver function tests (LFTs) were elevated, with an alkaline phosphatase of 515 IU/L, aspartate aminotransferase of 198 IU/L, and total bilirubin of 10.3 mg/dL. Carbohydrate antigen 19-9 (CA 19-9) at this time was 395 U/mL. Upon further workup by a gastroenterologist, endoscopic ultrasound (EUS) with fine needle aspiration (FNA) revealed adenocarcinoma of the pancreatic head in addition to an incidental 2.0 cm distal esophageal exophytic lesion that returned positive for adenocarcinoma. The relationship of these two carcinomas was uncertain. Endoscopic retrograde cholangiopancreatography (ERCP) was also performed for metallic biliary stent placement to relieve high-grade biliary obstruction related to the pancreatic mass.

Further imaging with 18-fluorodeoxyglucose positron emission tomography (FDG-PET)/CT demonstrated a large hypodense mass in the head of the pancreas with moderate FDG activity consistent with the patient’s known PDAC in addition to multiple enlarged peripancreatic, aortocaval, and porta hepatic lymph nodes as well as a focal area of mild metabolic activity in the distal esophagus just above the gastroesophageal junction with multiple paraesophageal lymph nodes. At an outside institution, the patient was diagnosed with localized PDAC that was thought to be unresectable along with a separate esophageal adenocarcinoma primary. Therefore, his local oncologist recommended chemotherapy with FOLFIRNOX (5-fluorouracil, leucovorin, irinotecan, and oxaliplatin) and referred the patient to our Pancreatic Multidisciplinary Clinic (PMDC) for additional recommendations. [14]

**Figure 1:**
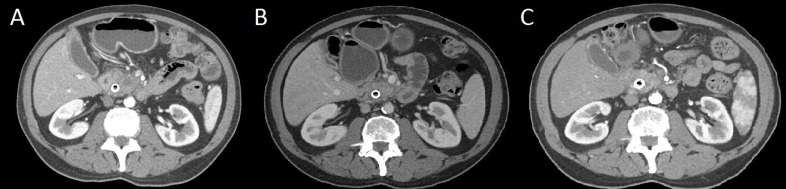
**A.** Pre-treatment computed tomography (CT) scan demonstrating ill-defined infiltrative mass measuring 2.7 cm x 5.0 cm**. B.** CT following 6 doses of FOLFIRINOX chemotherapy showing that the mass involving the head and uncinate process of the pancreas is difficult to define and measure but appears slightly less bulky as compared to the prior examination. **C.** 6-weeks post-SBRT CT scan reveals interval decrease in infiltrative pancreatic head mass.

Approximately one month from initial presentation, the patient was seen in our PMDC for a second opinion. Review of the outside pathologic slides confirmed moderately differentiated adenocarcinoma of the pancreas and adenocarcinoma in the distal esophagus; however, histologic distinction of the esophageal lesion as a primary tumor or metastasis was inconclusive. A repeat CT confirmed an ill-defined 2.7 x 5.0 cm mass within the pancreatic head/uncinate process of the pancreas invading into the 2^nd^ and 3^rd^ portions of the duodenum and demonstrating proximal main pancreatic duct dilation. Vessel involvement included encasement of the SMV/portal vein (PV) confluence and 180° abutment of the SMA, thereby conferring a diagnosis of borderline resectable PDAC (Figure 1A). CA 19-9 and hemoglobin A1C were elevated at this time at 315.1 U/mL and 6.4%, respectively, while carcinoembryonic antigen (CEA) was within normal range (2.4 ng/mL).

Suspecting borderline resectable PDAC and an early-stage esophageal primary, our multidisciplinary team recommended neoadjuvant chemotherapy followed by standard chemoradiation (CRT) or stereotactic body radiation therapy (SBRT) with re-evaluation for potential surgical resection. FOLIFIRINOX was the recommended chemotherapy such that the platinum agent would have activity in both primary pancreatic and esophageal tumors. Depending on the expertise of the thoracic oncologists and tumor response to chemotherapy, standard CRT would be warranted in order to encompass both the esophagus and pancreas in the same field; however, if the esophageal lesion would not require neoadjuvant radiation, SBRT to the pancreas lesion would be preferred. In order to address the suspected esophageal lesion, our thoracic colleagues were consulted and the patient was referred for formal evaluation by a thoracic surgeon.

After endoscopy and thoracic surgical consultation, the esophageal lesion was thought to be a synchronous esophageal primary cancer (T1bN1Mx, with no dysphasia symptoms) and the treatment recommendation consisted of neoadjuvant FOLFIRINOX followed by SBRT and evaluation for surgery. It was understood that treatment of the PDAC was of primary significance, with the possibility of delivering definitive CRT to the esophagus later in the treatment course.

### Neoadjuvant therapy

The following week, FOLFIRINOX was initiated locally and continued for 3 months (notably, irinotecan was held for the first 2 doses due to elevated LFTs). Following 6 doses of FOLFIRINOX, the patient presented back to our PMDC for re-evaluation. The patient continued to work 12-hour days throughout therapy, with his only complaint being minor fatigue. His CA 19-9 had decreased to 71.9 U/mL at this time (4 months from diagnosis), with CT demonstrating the pancreatic mass and regional lymphadenopathy to be slightly less bulky, improvement of SMA/SMV involvement (Figure 1B), and improved visualization of the esophageal thickening. Our multidisciplinary team recommended 2 additional months of FOLFIRINOX followed by SBRT if no disease progression and re-evaluation for surgery and/or irreversible electroporation (IRE). The patient resumed chemotherapy and received 6 additional doses, for a total of 12 doses of FOLFIRINOX over 6 months.

**Figure 2:**
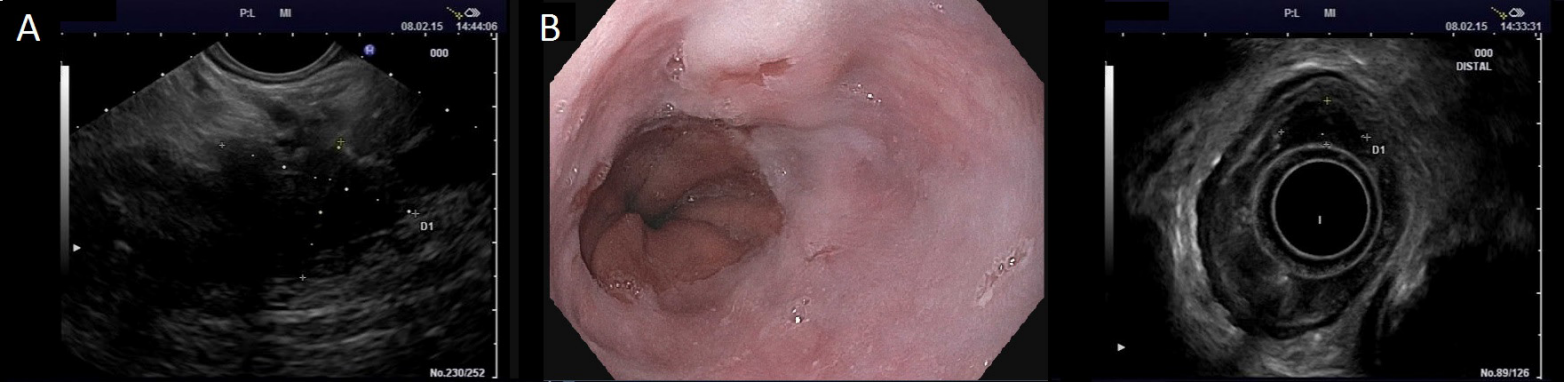
Visualization of the A. pancreatic lesion on endoscopic ultrasound (EUS) and B. esophageal lesion on endoscopy and EUS at the time of fiducial placement prior to SBRT

The patient then underwent SBRT to the pancreatic tumor to a total cumulative dose of 30.5 Gy in 5 fractions. Image guidance was performed using 3 gold fiducial markers endoscopically placed around the lesion and active breathing control (ABC) was used to minimize movement of the tumor during respiration. Images of the pancreatic and esophageal lesions at the time of endoscopy can be visualized in Figure 2. The patient’s only complaint during SBRT was mild (grade 1) fatigue. Three weeks after the completion of SBRT, CT imaging showed a slight interval decrease in the infiltrative pancreatic head mass and regional lymphadenopathy without definite evidence of vascular invasion (Figure 1C). CA 19-9 further decreased to 41.7 U/mL, nearly an 8-fold decrease from diagnosis. The patient was considered a surgical candidate at this time, with the plan to proceed forward with a combined approach of pancreaticoduodenectomy and esophagectomy to remove both the pancreas and esophageal tumors, respectively, in four weeks.

Of note, an esophagogastroduodenoscopy (EGD) was performed at the time of endoscopic fiducial placement to re-biopsy the esophageal lesion. The morphology was most consistent with a carcinoma that spread from the pancreaticobiliary system and immunolabeling for SMAD4 demonstrated retention of labeling, which neither confirmed nor refuted an interpretation of spread from a pancreaticobiliary lesion. The patient also experienced a few episodes of hematochezia during chemotherapy. A colonoscopy was performed and reported as negative, with the bleeding resolving spontaneously.

### Surgical resection

**Figure 3:**
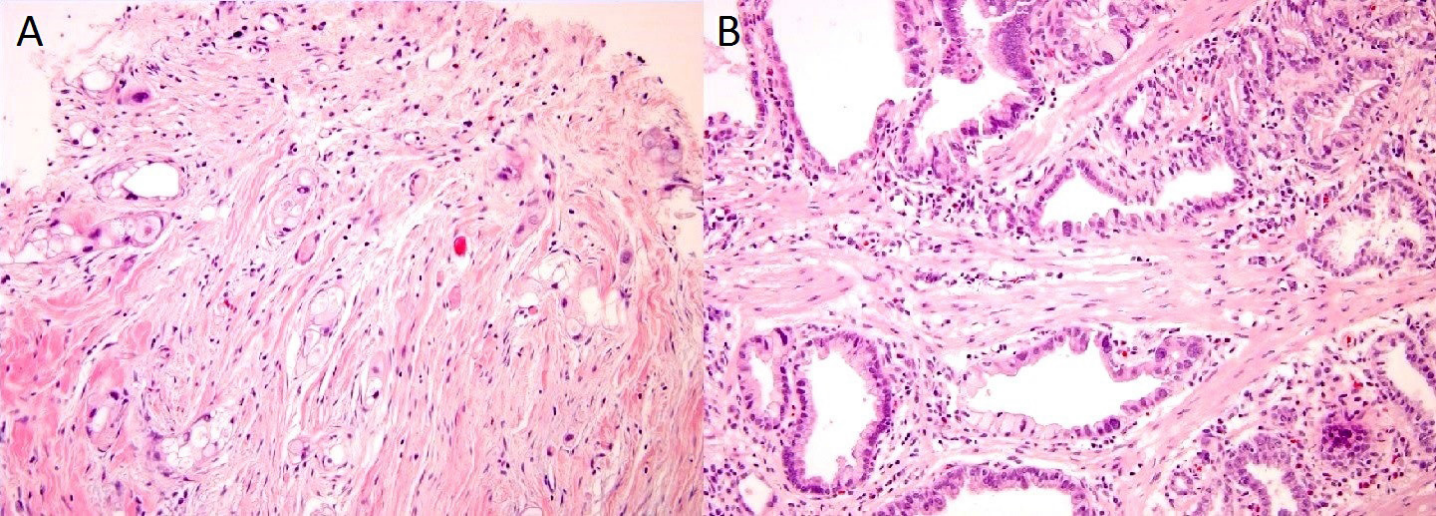
Evidence of fibrosis in the pancreatic primary A. and esophageal B. specimen

**Figure 4:**
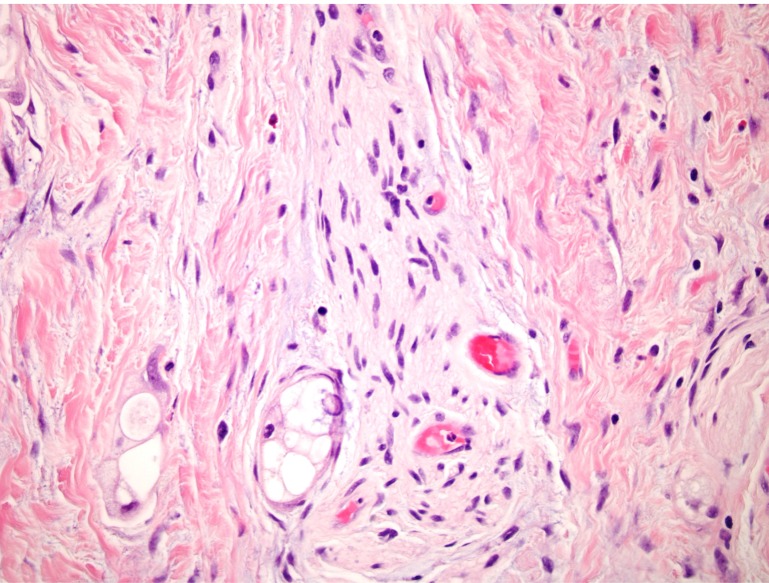
Evidence of perineural invasion of the pancreatic primary

Eight months after initial diagnosis and after six months of neoadjuvant therapy, the patient underwent a pylorus-preserving pancreaticoduodenectomy and Ivor Lewis esophagectomy with jejunostomy feeding tube (J-tube) placement. During the operation, the right gastric artery was preserved and the blood supply to the stomach was confirmed both visually and with an intraoperative Doppler ultrasound. The pancreatic specimen revealed numerous microscopic foci of adenocarcinoma with vacuolated cytoplasm and hyperchromatic nuclei scattered within a 5 cm fibrotic tumor bed (Figure 3A), otherwise defined as a near pathologic complete response to neoadjuvant therapy. Despite the minimal residual invasive carcinoma and extensively fibrotic background, it was considered a moderate response to neoadjuvant therapy due to the number of foci present and the area across which they were dispersed. A successful margin-negative resection was achieved, with a distance of invasive carcinoma 4 mm to the uncinate margin; however, 3 of 16 lymph nodes contained metastatic carcinoma, with the largest tumor focus being 3 mm. Local extension to the wall of duodenum and lymphovascular invasion were present, as was perineural invasion (Figure 4).

**Figure 5:**
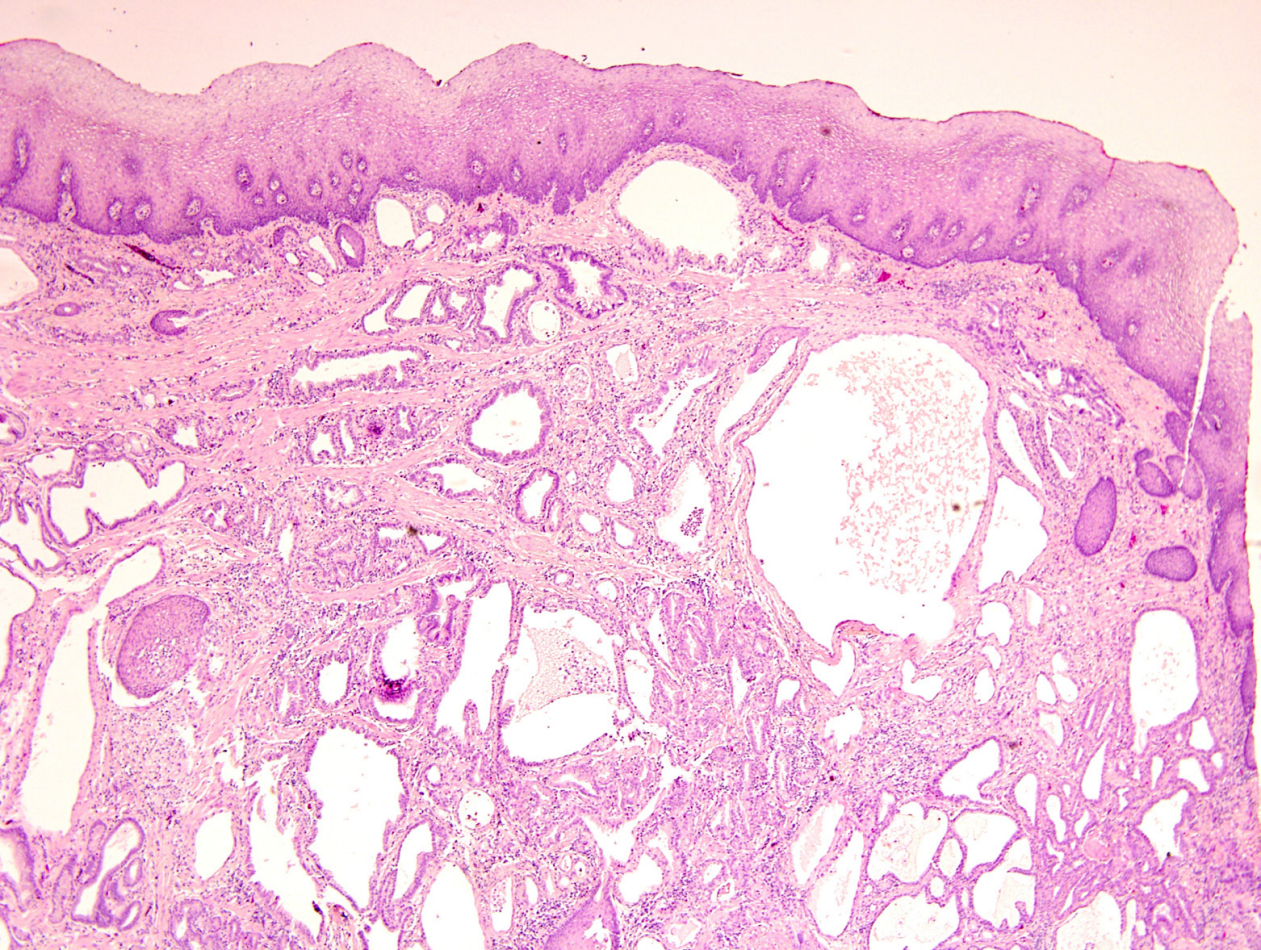
The lack of *in situ* component in the overlying epithelium provides supporting evidence that the suspected primary esophageal lesion was actually an esophageal metastasis from PDAC

The esophageal specimen revealed a microscopic focus (3 mm) of infiltrating adenocarcinoma involving submucosa of the distal esophagus (Figure 3B) along with an incidental leiomyoma 4 mm in size. Resection margins were uninvolved and all 14 lymph nodes were negative for tumor. Focal Barrett mucosa of the distinctive type with Paneth cell metaplasia was observed with no evidence of high-grade dysplasia or an *in situ* carcinoma. Focally active chronic gastritis and chronic cholecystitis were noted. The normal overlying squamous mucosa as shown in Figure 5 is strong evidence that the esophageal lesion represents a metastasis from the patient’s primary PDAC. Therefore, the final pathologic diagnosis was yT3yN1yM1 PDAC.

### Follow-up

There were no major surgical complications although gram-positive cocci in chains were found in the wound and treated with antibiotics and negative-pressure wound therapy was performed for drainage at the incision site. One month from surgery, the patient was doing well with no major complaints other than intermittent abdominal pain. His J-tube was removed at the time of two month follow-up and adjuvant chemotherapy was recommended; however, numerous appointments were missed and the patient refused adjuvant therapy. Unintentional weight loss secondary to pain during eating and acid reflux led to a nutrition consultation and prescription of a proton pump inhibitor and pancreatic enzyme supplementation. The patient occasionally complains of abdominal pain and nausea but is doing well otherwise and remains with no evidence of disease 17 months from surgery and 25 months from diagnosis, with surveillance laboratory studies and imaging occurring every 3 months.

## DISCUSSION

This case report summarizes a unique case of a patient with PDAC with an isolated metastasis to the esophagus who underwent successful resection of a pancreatic head and distal esophageal lesion after neoadjuvant therapy. To our knowledge, this is the 12^th^ reported case of an isolated esophageal metastasis from PDAC and the 2^nd^ report on an aggressive approach of a combined pancreaticoduodenectomy and esophagectomy. [7-13, 15]

The patient’s pancreatic primary showed moderate treatment effect among a background of extensive fibrosis, whereas the esophageal lesion showed a more classic appearance of an untreated adenocarcinoma with numerous dilated glands with an infiltrative growth pattern, nuclear atypia, and scattered mitotic figures. While there was focal Barrett’s mucosa elsewhere in the esophagus, it did not show significant dysplasia. Most significantly, the adenocarcinoma in this case had overlying squamous mucosa with no significant histopathologic change. While SMAD4 expression was intact in the esophageal lesion, roughly half of PDACs retain SMAD4 expression, and this result does not exclude spread from a PDAC primary. [16, 17]

This case report elucidates the difficulty of differentiating an esophageal metastasis from PDAC primary *versus* a synchronous esophageal carcinoma. Despite numerous methods of imaging and procedures including CT, PET/CT, EUS, EGD, and immunohistochemistry as well as multidisciplinary review among radiology, pathology, gastroenterology, surgery, medical oncology, and radiation oncology at a high-volume tertiary center, the final diagnosis of metastatic PDAC to the esophagus was not reached until a substantial amount of tissue was reviewed after partial esophagectomy. FDG-PET is commonly used in combination with CT and/or EUS to identify occult metastases in pancreatic and esophageal adenocarcinomas. However, the sensitivity and specificity of detection of metastases range from 50-90% and, as observed in this case report, may not lead to conclusive evidence. [18-21]

In general, local therapy is not traditionally recommended for metastatic disease, PDAC or otherwise; however, oncologists are becomingly increasingly aggressive by offering radiation therapy and/or surgical resection in this patient population, particularly in the setting of limited oligometastatic disease. [5, 22-27] In fact, surgical resection of metastases to the esophagus from distant organs has historically been reported as a promising and viable option for cases in which the primary tumor growth rate is suspected to be slow. [5] Although the morbidity and mortality associated with major operations such as a pancreatectomy and esophagectomy may be expected to be high, outcomes have improved tremendously in recent decades, especially with surgeons who are experienced and operate on a large volume of patients annually. [28, 29] Notably, combining an esophagectomy with a pancreaticoduodenectomy requires advanced planning and efficient coordination between both thoracic and hepatobiliary surgeons. A short course of radiation therapy with SBRT may also be a reasonable option to maximize local control with very little toxicity in oligometastatic PDAC, [24, 26, 27, 30] especially in cases in which a break from systemic therapy is necessary due to intolerability.

Neoadjuvant FOLFIRINOX was administered in this patient as a method to provide aggressive systemic therapy and include a platinum agent that may warrant a treatment response in both the PDAC and esophageal lesion. In 2011, Conroy and colleagues published the results of a randomized clinical trial comparing FOLFIRINOX and gemcitabine monotherapy. [31] FOLFIRINOX was superior to gemcitabine in terms of overall survival (11.1 months *vs*. 6.8 months), progression-free survival (6.4 months *vs*. 3.3 months), and objective response (31.6% *vs*. 9.4%). Since then, FOLFIRINOX has been studied in other settings of PDAC as well as other gastrointestinal cancers, with promising response rates in patients who are able to tolerate the regimen. [32-35]

An esophageal metastasis from a pancreatic primary may be more common than traditional belief and patients with a suspicious esophageal lesion should undergo comprehensive evaluation and close follow-up in order to guide management. Although there are limited data to suggest an optimal approach to these cases, neoadjuvant FOLFIRNOX followed by SBRT and surgery has resulted in favorable disease control over two years from diagnosis despite no adjuvant therapy. However, we only recommend aggressive surgery of both lesions if there is no clear evidence of metastatic disease at other locations after an extended period of time ( > 6 months). As future technologies involving novel analytic techniques such as next-generation sequencing, [36, 37] circulating tumor DNA, [38, 39] and intravital microscopy [40] advance, oncologists will be more likely to predict treatment response and make improved treatment recommendations accordingly.

## MATERIALS AND METHODS

After obtaining consent from the patient, all relevant records of the case were retrospectively reviewed. The pathologic specimens were reviewed with response to neoadjuvant therapy graded using the criteria described previously by the College of American Pathologists. [41]

## Abbreviations

(PDAC): Pancreatic ductal adenocarcinoma
(CT): Computed tomography
(SMA): Superior mesenteric artery
(SMV): Superior mesenteric vein
(LFTs): Liver function tests
(CA 19-9): Carbohydrate antigen 19-9
(EUS): Endoscopic ultrasound
(FNA): Fine needle aspiration
(ERCP): Endoscopic retrograde cholangiopancreatography
(FDG-PET): 18-fluorodeoxyglucose positron emission tomography
FOLFIRINOX: (5-fluorouracil, leucovorin, irinotecan, and oxaliplatin)
(PMDC): Pancreatic Multidisciplinary Clinic
(PV): Portal vein
(CEA): Carcinoembryonic antigen
(CRT): Chemoradiation
(SBRT): Stereotactic body radiation therapy
(IRE): Irreversible electroporation
(ABC): Active breathing control
(EGD): Esophagogastroduodenoscopy
(J-tube): Jejunostomy feeding tube
